# Role of the National Immunisation Technical Advisory Groups in 13 European countries in the decision-making process on vaccine recommendations

**DOI:** 10.2807/1560-7917.ES.2023.28.43.2300131

**Published:** 2023-10-26

**Authors:** Domenico Martinelli, Filippo Quattrone, Francesca Fortunato, Elisa Di Maggio, Antonietta Filia, Maria Cristina Rota, Pier Luigi Lopalco, Rosa Prato

**Affiliations:** 1 Hygiene Unit, Policlinico Foggia Hospital, Department of Medical and Surgical Sciences, University of Foggia, Foggia, Italy; 2 Management and Healthcare Laboratory, Institute of Management and Department EMbeDS, Scuola Superiore Sant'Anna, Pisa, Italy; 3 Department of Infectious Diseases, Istituto Superiore di Sanità, Rome, Italy; 4 Department of Biological and Environmental Sciences and Technology, University of Salento, Lecce, Italy

**Keywords:** NITAG, immunisation schedule, vaccines, vaccine-preventable diseases, EU-JAV

## Abstract

In Europe, National Immunisation Technical Advisory Groups (NITAGs) were established in most countries to promote evidence-informed decision-making in introducing new or improved vaccines or changing recommendations for existing ones. Still, the role, activities and outcomes of NITAGs have not been optimally implemented across Europe. Within the European Joint Action on Vaccination (EU-JAV), we conducted a survey to collect information on decision-making process including the main criteria for the introduction of new vaccines or changes to recommendations on their use. Between December 2021 and January 2022, 13 of the 28 European countries invited participated in an online survey. The criteria ranked as most relevant were disease burden and availability of financial resources. Only one country specified that the NITAG recommendations were binding for the government or the health authority. Vaccinations more often reported for introduction or recommendation changes were those against herpes zoster, influenza, human papillomavirus infection, pneumococcal and meningococcal disease. The planned changes will mainly address children and adolescents (2–18 years) and adults (≥ 45–65 years). Our findings show potential overlaps in the activities of NITAGs between countries; and therefore, collaboration between NITAGs may lead to optimisation of the workload and better use of resources.

## Background

Introducing new evidence-based and economically sustainable vaccine products and new technologies into national immunisation programmes is essential to adapt to a continuously evolving landscape. Such landscape is characterised by the introduction of new preventive measures against emerging pathogens and a progressive evolution of vaccination programmes towards life course immunisation and protection of populations at risk. The National Immunization Technical Advisory Groups (NITAGs) are critical actors in ensuring continuous updates of national immunisation strategies [1,2]. According to the World Health Organization (WHO) recommendations on adding a vaccine to a national immunisation programme [1], NITAGs are responsible for providing independent, well-informed advice to the national government bodies based on a thorough review of the available evidence. Before recommending introduction of a vaccine, NITAGs should consider several factors: the disease, including its burden, public health or political priorities, availability of other preventive and control measures, the vaccine, including the efficacy and safety, economic and financial aspects, supply availability, strength of the national immunisation programme and the health system to accommodate the vaccine [1,3]. According to Strategic Advisory Group of Experts on Immunization (SAGE) standards for the development of evidence-based vaccination-related recommendations, additional criteria such as equity and acceptability of the intervention should be considered [4]. Moreover, policymakers and expert advisory committee members increasingly value the interventions based on economic evaluations, as recently shown by a systematic review on criteria for decision-making on vaccine adoption [5]. Nevertheless, a survey performed by the European Centre for Disease Prevention and Control (ECDC) in 2015 highlighted a variety of roles in the decision-making process and the different theoretical frameworks used by the NITAGs of the European Union/European Economic Area (EU/EEA) countries to propose introduction of new vaccinations [6].

Implementation of NITAGs has been a goal of the European Vaccine Action Plan (EVAP) 2015–2020 of the WHO Regional Office for Europe (WHO/Europe) that monitored the establishment of new NITAGs, the respect of WHO criteria for functionality and whether they provided reviews of relevant evidence to allow informed decisions on the introduction of a new vaccine [1,3]. According to the data from the WHO/the United Nations Children’s Fund (UNICEF) Joint Reporting Form on Immunization (JRF), in 2022, all WHO European Region countries or territories have an established NITAG except for Kosovo* [7]. Hungary has a committee composed of members from the National Institute of Public Health that acts as decision-support. As part of the EVAP 2015–2020, WHO European Region states were required to report on whether their NITAGs made recommendations for or against the introduction of three vaccines, namely pneumococcal conjugate vaccine (PCV), rotavirus vaccine or human papillomavirus (HPV) vaccine [3]. Not all NITAG recommendations in favour of a vaccine have led to an introduction, principally due to challenges related to vaccine availability and financial sustainability. The role of NITAGs and their centrality in updating the national immunisation schedule and optimising the public health impact of existing and newer vaccines has also been stressed in the strategic priorities of the European Immunisation Agenda 2030 [8].

## Scope and data collection

We performed an online survey entitled ’Introduction of new or improved vaccines and possible upcoming changes to recommendations for existing vaccines’ among NITAG representatives or persons in charge of the national or subnational immunisation programmes. The survey was developed within the European Joint Action on Vaccination (EU-JAV) of the European Union Health Programme (www.eu-jav.com). We aimed at collecting information about the main criteria for vaccine recommendation development in European countries and any upcoming plans to introduce new vaccine products (new vaccines or vaccine combinations) and/or new vaccine recommendations into national immunisation programmes during the years 2022-2024.

Between December 2021 and January 2022, 28 European countries were invited to participate (20 EU-JAV consortium partners and eight EU/EEA countries not participating in the EU-JAV). The email addresses were provided by mapping of NITAG representatives (i.e. NITAG chairs, members and secretariats) or persons in charge of the national or subnational immunisation programmes (i.e. representative of the Ministry of Health or National Health Institutes), created as a deliverable of the EU-JAV project (Work package 4, coordinated by the Italian National Health Institute). We verified the contact list using institutional websites. An online survey link was provided, accompanied by an information sheet detailing the purpose and data handling procedures of the study.

After a review of the literature and documents from international and national agencies [1,6,9], a questionnaire was developed, consisting of 10 multiple-choice and open-ended questions. The questionnaire was piloted for comprehensibility and answerability with the help of four expert reviewers in vaccinology. Information on the following topics were collected:

1. Country, name, affiliation and contact details of respondents

2. Key criteria that inform vaccine recommendation development of the country

3. New vaccine introduction and/or recommendations planned for 2022-2024, by target age group (infants and toddlers, children and adolescents, adults, elderly), by medical condition and other indications.

The questionnaire was created and administered via on online tool, Surveymonkey. Details of the survey questions are available in Supplementary material. The survey was followed by deeper phone discussions or email exchanges when necessary.

## Participating countries

Thirteen of the 28 invited countries responded to the survey: Belgium, Bosnia-Herzegovina, Croatia, Denmark, Hungary, Ireland, Italy, Latvia, Norway, Portugal, Romania, Spain and Sweden. Representatives of NITAGs responded on behalf of Ireland, Latvia and Portugal, NITAG Secretariats answered on behalf of Belgium, Norway and Spain, a representative of the Ministry of Health on behalf of Italy and representatives of the National Public Health Institutes answered for Bosnia-Herzegovina, Croatia, Denmark, Hungary, Romania and Sweden.

## Key criteria for vaccine recommendation development

All participants of the survey, hereafter referred to as countries, ranked the five key criteria for vaccine recommendation development in their countries. Compared with a previous NITAG survey showing that the range of approaches used by the various NITAGs within the vaccine recommendation process was large and differed between countries [10], participants in this survey ranked disease burden and availability of financial resources as the most relevant criteria that inform vaccine recommendation development (Figure). This appears in line with the principles and considerations proposed by the WHO in 2014 for deciding whether to introduce a vaccine into national immunisation programmes [1]. Notably, the least ranked option was the presence of alternative vaccines, while many new introductions or upcoming changes reported regarded the replacement of existing vaccines with improved ones. Therefore, it is possible that respondents did not consider improved vaccines as alternative ones or that they found this option not relevant in general. The other key criteria proposed received similar scores, highlighting that they were considered similarly relevant. This is coherent with the WHO advice to NITAGs to treat all criteria as essential for the decision-making process [11].

**FIGURE:**
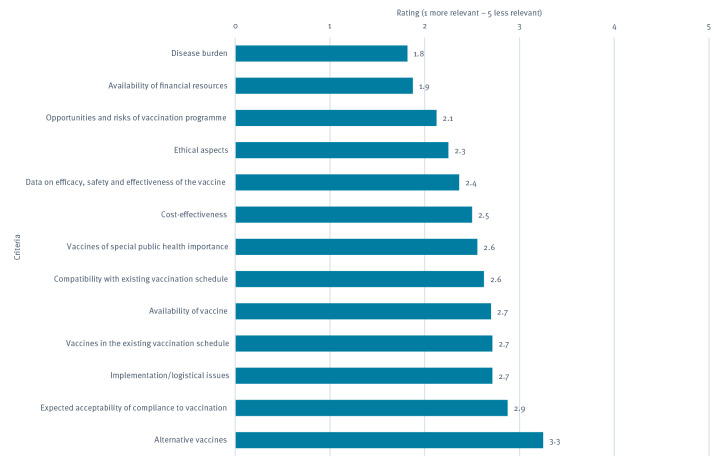
Criteria for development of vaccine recommendations in 13 European Union/European Economic Area countries

In all countries except Romania, where NITAG recommendations are binding for the government or the health authority, NITAGs have an advisory role, with the Ministry of Health or other authorities deciding on the recommendations. A prior survey indicated that separate government or health insurance entities often determine vaccine introductions or reimbursements [6]. Moreover, a NITAG can endorse a recommendation for a vaccine, but vaccine use may not be adopted, e.g. for economic reasons. In some cases, additional committees or authorities are responsible for decision-making at subnational level [12]. These findings support the idea that, besides the WHO Global Vaccine Action Plan 2020 goal of having a NITAG established in all countries, performance indicators of NITAG activity are also necessary [2]. Since 2010, there have been six functional indicators of a NITAG, assessed annually in the WHO/UNICEF JRF [13]. These indicators included establishment of a NITAG, presence of formal written terms of references, legislative foundation, member expertise, meeting regularity and preparatory practices. From 2021 onwards, the assessment expanded to encompass the timing of the most recent NITAG review, if the NITAG issued recommendations and if NITAG recommendations were adopted by the Ministry of Health [14]. Nevertheless, more sophisticated frameworks are required to better measure functional capacity, quality of NITAG processes and outputs and integration into national immunisation decision-making systems [15,16].

## New vaccine introduction and/or recommendations planned, 2022 to 2024

Only nine out of thirteen respondent countries (Belgium, Croatia, Denmark, Ireland, Italy, Latvia, Norway, Spain and Sweden) specified which vaccine introductions or recommendations have been planned for the period 2022-2024. Hungary and Romania reported not knowing whether new immunisations will be implemented. Portugal and Bosnia and Herzegovina gave no further information on which vaccinations they will introduce or update.

Age groups mostly targeted by planned updates of national immunisation plans were children and adolescents (aged 2–18 years) and adults (aged 45–65 years or older) (Table). This is in line with the progressive shift towards a life-course approach to immunisation from the traditional focus on infants only. Notably, only one country reported a new recommendation for healthcare workers (HCW), although COVID-19 pandemic has highlighted the importance of prevention of vaccine-preventable diseases and the feasibility of extensive vaccination campaigns in this population group [17]. Actually, all European countries have established vaccine recommendations for HCW [18], but policies remain heterogeneous with regard to targeted diseases, HCW groups and force of recommendation [19]. Lack of political and managerial commitment towards such important issue, that should also take into consideration compulsory vaccination, represents a gap worth addressing.

**Table:** Planned vaccine introductions or recommendations in 13^a^ European Union/European Economic Area countries, by target age group, other indications and disease, 2022-2024

Country	Number of planned vaccine introductions or recommendations													
	Target age group^b^ (years)			Individuals with medical conditions or other indications	Disease									
	< 2	2–18	45–≥ 65		Acellular pertussis	DTP	Herpes zoster	HPV infection	Meningitis B	Meningococcal disease	Pneumococcal disease	Rotavirus infection	Seasonal influenza	Varicella
Belgium	3	1	1	0	0	0	1	0	1	2	0	0	1	**0**
Croatia	0	0	0	1			1		0	0				
Denmark		1	0	2			0	1					2	
Ireland	2	1	1	1			2	0					0	2
Italy	2	2	2	2			2	1	1	2	1		1	0
Latvia	0	2	0	1	1	1	0	1	0	0	0		0	
Norway		2	2	0	0	0	1	0	0	1	1			1
Spain	1	3	1	0			0	1	1	0	1	1	1	0
Sweden	0	0	1	0				0	0		1	0	0	
Total	8	12	8	7	1	1	7	4	3	5	4	1	5	3

DTP: diphtheria-tetanus-pertussis; HPV: human papillomavirus.

^a^ No vaccine introductions or recommendations planned in Bosnia-Herzegovina, Hungary, Portugal and Romania.

^b^ No country had vaccine introductions or recommendations planned for age group 19-44 years.

According to our survey, planned vaccine introductions or recommendations were most common against herpes zoster, influenza, HPV infection, pneumococcal disease and meningococcal disease (Table, Box). Herpes zoster vaccination is currently part of the immunisation programmes of seven EU countries (Austria, Czech Republic, Estonia, France, Germany, Greece and Italy) [9,20]. Changes to existing recommendations for herpes zoster vaccination in adults or individuals with underlying medical conditions are planned in Belgium, Croatia, Ireland, Italy and Norway. In particular, Belgium and Ireland are planning to shift to the recombinant zoster vaccine, as has already been done in other countries (i.e. the United States since December 2020 and New Zealand since August 2022) [21,22]. Belgium, Denmark, Italy and Spain will have new recommendations for seasonal influenza vaccination for healthy toddlers and younger children. Some European countries, including Finland, Latvia, Slovakia [9] and the United Kingdom (UK), have already initiated influenza immunisation programmes for these younger age groups based on the evidence of significant effects of the childhood immunisation programme on the reduction of influenza virus circulation among children and possible indirect benefits to older age groups [23,24]. Extension of HPV vaccination using the nonavalent formulation is planned in four countries, moving to a gender-neutral approach in Latvia and Spain, as lately done in 19 EU/EEA countries (Austria, Belgium, Croatia, Czech Republic, Denmark, Finland, France, Germany, Hungary, Ireland, Italy, Luxembourg, the Netherlands, Norway, Poland, Portugal, Slovakia, Slovenia and Sweden) [25]. In Denmark, vaccination against HPV will be offered also to men who have sex with other men, as has been done in the UK and Italy, and to women with cervical intraepithelial neoplasia (CIN) grade 2 or higher in Italy. Introduction or update of pneumococcal vaccines was proposed in four countries, reflecting the accumulating evidence of the use of pneumococcal conjugate vaccines in the elderly and the upcoming new formulations (15 and 20 valent PCV) [26]. The introductions of vaccination against meningococcal B disease in infants (Belgium and Spain), coherently with ECDC recommendations [27], and the switch from the monovalent meningococcal C vaccine to the tetravalent meningococcal vaccine (Belgium, Italy and Norway) are planned. Even if immunisation policies of 10 EU/EEA countries (Austria, Czech Republic, France, Greece, Ireland, Italy, Lithuania, Luxembourg, Malta and Portugal) entail vaccinations of infants against meningococcal B disease [9], still, recommendations for meningococcal B vaccination are highly heterogeneous across countries. The switch to the tetravalent meningococcal vaccine has already been done in the immunisation plans of five European countries (Belgium, Czech Republic, Greece, Malta and the Netherlands) [9], driven by accumulating evidence and the WHO Global Roadmap [28].

Box **Planned vaccine introductions or recommendations in 13^a^ European Union/European Economic Area countries, 2022-2024**

**Belgium:**

• Recommendation for meningococcal vaccination of infants (birth–12 months) with meningococcal B vaccine

• Recommendation for meningococcal vaccination of toddlers (13–24 months) and adolescents (11–18 years) with ACWY conjugate vaccine (instead of meningococcal C vaccine)

• Recommendation for seasonal influenza vaccination of infants (from 6 months) with quadrivalent vaccine

• Recommendation for herpes zoster vaccination of adults ≥ 65 years with recombinant, adjuvanted vaccination

**Croatia:**

• Recommendation for herpes zoster vaccination of persons with underlying medical conditions

**Denmark:**

• Recommendation for seasonal influenza vaccination of children (2–6 years) with live-attenuated, egg-based vaccine

• Recommendation for seasonal influenza vaccination of healthcare workers with inactivated, standard-dose, egg-based vaccine

• Recommendation for HPV vaccination of men who have sex with men with nonavalent vaccine^b^

**Ireland:**

• Review of the vaccination schedule of infants (birth–12 months)

• Recommendation for varicella vaccination of toddlers (13-24 months) and children (2–10 years)

• Recommendation for herpes zoster vaccination of adults (≥ 60–65 years) and persons with underlying medical conditions with recombinant, adjuvanted vaccine

**Italy:**

• Recommendation for meningococcal B vaccination of infants and toddlers (birth–24 months) using reduced schedule of two primary doses and a booster dose

• Recommendation for seasonal influenza vaccination of children (6 months–6 years) with quadrivalent vaccine

• Recommendation for meningococcal vaccination of toddlers (13-24 months) and adolescents (11-18 years) with ACWY conjugate vaccine (instead of meningococcal C vaccine)

• Recommendation for pneumococcal and herpes zoster vaccination of adults (≥ 65 years)

• Recommendation for herpes zoster vaccination of persons with underlying medical conditions using recombinant, adjuvanted vaccination

• Recommendation for HPV vaccination of women with CIN grade ≥ 2 with nonavalent vaccine

**Latvia:**

• Introduction of gender-neutral HPV vaccination of adolescents (11-18 years)

• Introduction of DTP vaccination of adolescents (11-18 years)

• Introduction of pertussis vaccination of pregnant women

**Norway:**

• Recommendation for varicella vaccination of toddlers (13-24 months) (under consideration)

• Recommendation for meningococcal vaccination of adolescents (11-18 years) (under consideration)

• Recommendation for pneumococcal and herpes zoster vaccine of the elderly (≥60-65 years)^c^

**Spain:**

• Introduction of rotavirus vaccination in infants^d^

• Introduction of meningococcal B and seasonal influenza vaccination of children^d^

• Introduction of HPV vaccination of male adolescents^d^

• Introduction of new pneumococcal vaccines of adults^d^

**Sweden:**

• Introduction of pneumococcal vaccination in a national programme for elderly (≥65 years)

ACWY: serogroups A, C, W and Y; HPV: human papillomavirus; CIN2 +: cervical intraepithelial neoplasia grade ≥ 2; DTP: diphtheria-tetanus-pertussis.
^a^ No vaccine introductions or recommendations planned in Bosnia-Herzegovina, Hungary, Portugal, and Romania.
^b^ Introduced as a temporary programme.
^c^ Health technology assessment and health economic evaluation are in progress before to final advice is given to the Ministry of Health.
^d^ Age not reported.

## Discussion

This study evaluated the role of European NITAGs in updating national immunisation plans covering the introduction of new or improved vaccines and recommendation changes. Although the response rate (46%; 13/28) of the survey limits its generalisability across Europe, the results give insights into planned vaccine introductions, complementing tools like the Vaccine Scheduler of the ECDC [9], which includes new vaccinations only when they are already introduced in national immunisation plans. A cross-check of the ECDC vaccination tracker with the information from the present survey found that 15 (42.9%) new introductions or changes were already present in the tracker as of September 2023 (when we were finalising this manuscript). Moreover, the survey offered insights into the activities of European NITAGs: updated versions of the questionnaire could further characterise their functioning, collecting more in-depth information and ensuring that they comply with the European Immunization Agenda 2030 recommendations or other regulatory/evidence frameworks [8].

We included in our respondent pool NITAG experts and secretariats and persons in charge of the national or subnational immunisation programmes. While this increased country participation, it may have introduced variability in our findings. The survey did not explore the role of NITAGs in the introduction of vaccines during health emergencies (e.g. COVID-19 pandemic or mpox outbreak). This topic warrants further discussion, as these vaccinations are likely to be progressively integrated into the national immunisation programmes. Another limitation of this study was that NITAG-related documents such as recommendations or standard operating procedures were not reviewed to assess the criteria used in the evidence-to-recommendation process or overlapping among NITAGs activities.

The findings of this work highlight a fragmented scenario, where NITAGs are working autonomously with a risk of duplicating efforts or not providing equal access to vaccinations across all European countries despite similar disease burden in most countries [29]. This reflects that, even if almost all European countries have NITAGs in place, they have varying lengths of experience, with some NITAGs only recently been established [34]. Our observed overlap in NITAG recommendations differs from a previous five-country study with minimal overlap in issues and processes. This difference could stem from our broader country sample and our emphasis on new vaccine introductions, while in the period assessed by the previous study, no new vaccinations were included in the national immunisation plans of any of the five countries, i.e. there were fewer opportunities for NITAGs to conduct common activities [30].

## Conclusions

Decisions on vaccines and their use in immunisation schedules remain in the competence of each country, needing to be adapted to the national context. However, initiatives such as the EU/EEA NITAG collaboration [31] promote peer-to-peer partnership and the efficiency of scientific evidence collation. Stronger collaboration in the introduction of new vaccines among NITAGs, but also between NITAGs and other EU/EEA competent authorities and their networks (e.g. ECDC, European Medicines Agency) can be helpful in increasing manufacturing and supply capacity of vaccines and allowing dose-sparing strategies to ensure broader availability of immunisation products, as recently envisaged to counter global HPV vaccine shortage [32]. The COVID-19 vaccine introduction showcased robust coordination between organisations like WHO SAGE, ECDC and national NITAGs, promoting real-time knowledge sharing and evidence-based practices. Future vaccine introductions can leverage lessons from the COVID-19 experience [33]. Initiatives such as this survey can help assessing ongoing activities and promote synergy and knowledge transfer among European NITAGs in the core activity of supporting the continuous strengthening of national vaccination programmes.

## *Note

This designation is without prejudice to positions on status, and is in line with United Nations Security Council Resolution 1244/99 and the International Court of Justice Opinion on the Kosovo Declaration of Independence.

## Supplementary Data

787000 Supplementary Material
