# Supplementary material for: Role of the National Immunisation Technical Advisory Groups in 13 European countries in the decision-making process on vaccine recommendations
Source: Euro Surveill. 2023 Oct 26;28(43):2300131. doi: 10.2807/1560-7917.ES.2023.28.43.2300131 (PMC10604541; doi:10.2807/1560-7917.ES.2023.28.43.2300131)
Supplement: Supplementary Material [file 23-00131_MARTINELLI_Supplement.pdf]

This supplementary material is hosted by *Eurosurveillance* as supporting information alongside the article " Role of the National Immunisation Technical Advisory Groups in 13 European countries in the decision-making process on vaccine recommendations", on behalf of the authors, who remain responsible for the accuracy and appropriateness of the content. The same standards for ethics, copyright, attributions and permissions as for the article apply. Supplements are not edited by *Eurosurveillance* and the journal is not responsible for the maintenance of any links or email addresses provided therein.

Supplementary material 1

Questionnaire: EU-JAV: NITAG online survey on "Introduction of new or improved vaccines and possible upcoming changes to recommendations for existing vaccines"

Dear NITAG representative:

We would be grateful for your participation in this short online survey "Introduction of new or improved vaccines and possible upcoming changes to recommendations for existing vaccines", a task of the ongoing EU Joint Action on Vaccination (EU-JAV).

The aim of the survey is to collect information about upcoming plans to introduce new vaccine products (new vaccines or vaccine combinations) and/or new vaccine recommendations into the EU/EEA countries national immunization program during the next three years. You will be asked to complete the survey to gather in information regarding:

- *Your contact details, in case we need any further clarification or information*
- *Key criteria that inform vaccine recommendation development in your country.*
- *The new vaccine introduction and/or recommendations planned in the upcoming three years, by target age (infants and toddlers, children and adolescents, adults, elderly), by medical condition and other indications.*

Your participation in this study is your decision and you will not be adversely affected in any way if you choose not to participate. Once you have submitted the questionnaire, the data cannot be retracted. Your survey responses will be kept confidential and will not be linked to your name or contact information. Your name and contact information will be stored on a secure server and will not be shared or published.

Results from the survey will be summarised into a report that will be sent to you for your revision. Should you have any questions regarding the survey, please do not hesitate to contact us at any time at: domenico.martinelli@unifg.it, elisa.dimaggio@unifg.it.

Link to the questionnaire: https://it.surveymonkey.com/r/9H9LYBP

Thank you very much in advance for participating in this survey.

Kind regards,

EU-JAV WP6 Team - Italy

NITAG online survey on "Introduction of new or improved vaccines and possible upcoming changes to recommendations for existing vaccines"

Please provide your country, name, affiliation, and contact details

Country:	*Drop down menu*
Name of Respondent:	
Affiliation:	
Qualification and Role:	
E-mail:	
Telephone N:	

Q.1 - What is your current role in immunization in your country? (Please check all that apply)

[ ] National Immunization Technical Advisory Group (NITAG) representative

[ ] NITAG Secretariat

[ ] Ministry of Health

[ ] National Public Health Institute/equivalent

[ ] Other, specify ______________________________

Q.2 – Which key criteria inform vaccine recommendation development in your country? Please rank in a scale from 1 to 5 the five most relevant items (1 more relevant – 5 less relevant)

[ ] Disease burden

[ ] Availability of vaccine

[ ] Data on efficacy, safety, and effectiveness of the vaccine

[ ] Alternative vaccines

[ ] Vaccines of special public health importance

[ ] Cost-effectiveness

[ ] Opportunities and risks of vaccination programme

[ ] Expected acceptability ad adherence to vaccination

[ ] Compatibility with existing vaccination schedule

[ ] Vaccines in the existing vaccination schedule

[ ] Availability of financial resources

[ ] Implementation/logistical issues

[ ] Ethical aspects

[ ] Other, specify ____________________________

Q.3 - In your country, are NITAG recommendations "binding" for the government/health authority (i.e., must be implemented - e.g., a recommended new vaccine must be added to the immunization program schedule and the program implemented by the public authority)?

[ ] Yes

[ ] No

[ ] Do not know

Q.4 - Over the next three years, are you planning to introduce new vaccines and/or new vaccine recommendations into your national immunization program?

[ ] Yes

[ ] No

[ ] Do not know

*If **yes**: Please check all that apply*

Co-funded by
the Health Programme
of the European Union

Vaccine introduction / recommendations planned in the upcoming three years, by target age

Infants and toddlers

		Birth	Months																							Considerations	
Antigen			1	2	3	4	5	6	7	8	9	10	11	12	13	14	15	16	17	18	19	20	21	22	23	Introduced	Recommended
BCG																											
Hepatitis B																											
Polio																											
DTP-containing vaccine																											
Haemophilus influenzae type b																											
Pneumococcal	Conjugate																										
	Polysaccharide																										
Rotavirus																											
Meningococcal	MenA conjugate																										
	MenC conjugate																										
	MenACWY conjugate																										
	MenB																										
Measles, Mumps, Rubella																											
Measles, Mumps, Rubella, Varicella																											
Varicella																											
Hepatitis A																											
HPV	Nonavalent																										
	Quadrivalent																										
	Bivalent																										
Seasonal Influenza	Quadrivalent																										
	Trivalent																										
	Inactivated, standard-dose, egg-based																										
	Inactivated, standard-dose, cell culture-based																										
	Live attenuated, egg-based																										
	Inactivated, high-dose, egg-based																										
	Inactivated, standard-dose, egg-based with MF59 adjuvant																										
	Recombinant HA																										
Herpes zoster	Live, attenuated																										
	Recombinant, adjuvanted																										
Japanese Encephalitis																											
Yellow Fever																											
Tick-borne Encephalitis																											
Typhoid																											
Cholera																											
Rabies																											
Dengue (CYD-TDV)																											

:

Co-funded by
the Health Programme
of the European Union

Children and Adolescents

		Years																	Considerations	
**Antigen**		**2**	**3**	**4**	**5**	**6**	**7**	**8**	**9**	**10**	**11**	**12**	**13**	**14**	**15**	**16**	**17**	**18**	**Introduced**	**Recommended**
BCG																				
Hepatitis B																				
Polio																				
DTP-containing vaccine																				
Haemophilus influenzae type b																				
Pneumococcal	Conjugate																			
	Polysaccharide																			
Rotavirus																				
Meningococcal	MenA conjugate																			
	MenC conjugate																			
	MenACWY conjugate																			
	MenB																			
Measles, Mumps, Rubella																				
Measles, Mumps, Rubella, Varicella																				
Varicella																				
Hepatitis A																				
HPV	Nonavalent																			
	Quadrivalent																			
	Bivalent																			
Seasonal Influenza	Quadrivalent																			
	Trivalent																			
	Inactivated, standard-dose, egg-based																			
	Inactivated, standard-dose, cell culture-based																			
	Live attenuated, egg-based																			
	Inactivated, high-dose, egg-based																			
	Inactivated, standard-dose, egg-based with MF59 adjuvant																			
	Recombinant HA																			
Herpes zoster	Live, attenuated																			
	Recombinant, adjuvanted																			
Japanese Encephalitis																				
Yellow Fever																				
Tick-borne Encephalitis																				
Typhoid																				
Cholera																				
Rabies																				
Dengue (CYD-TDV)																				

Co-funded by
the Health Programme
of the European Union

Adults 19-44

		Years																										Considerations	
**Antigen**		**19**	**20**	**21**	**22**	**23**	**24**	**25**	**26**	**27**	**28**	**29**	**30**	**31**	**32**	**33**	**34**	**35**	**36**	**37**	**38**	**39**	**40**	**41**	**42**	**43**	**44**	**Introduced**	**Recommended**
BCG																													
Hepatitis B																													
Polio																													
DTP-containing vaccine																													
Haemophilus influenzae type b																													
Pneumococcal	Conjugate																												
	Polysaccharide																												
Rotavirus																													
Meningococcal	MenA conjugate																												
	MenC conjugate																												
	MenACWY conjugate																												
	MenB																												
Measles, Mumps, Rubella																													
Measles, Mumps, Rubella, Varicella																													
Varicella																													
Hepatitis A																													
HPV	Nonavalent																												
	Quadrivalent																												
	Bivalent																												
Seasonal Influenza	Quadrivalent																												
	Trivalent																												
	Inactivated, standard-dose, egg-based																												
	Inactivated, standard-dose, cell culture-based																												
	Live attenuated, egg-based																												
	Inactivated, high-dose, egg-based																												
	Inactivated, standard-dose, egg-based with MF59 adjuvant																												
	Recombinant HA																												
Herpes zoster	Live, attenuated																												
	Recombinant, adjuvanted																												
Japanese Encephalitis																													
Yellow Fever																													
Tick-borne Encephalitis																													
Typhoid																													
Cholera																													
Rabies																													
Dengue (CYD-TDV)																													

EU-JAV EUROPEAN JOINT ACTION ON VACCINATION

Co-funded by
the Health Programme
of the European Union

Adults 45-65 or older

		Years																							Considerations	
**Antigen**		**45**	**46**	**47**	**48**	**49**	**50**	**51**	**52**	**53**	**54**	**55**	**55**	**56**	**57**	**58**	**59**	**60**	**61**	**62**	**63**	**64**	**65**	**>65**	**Introduced**	**Recommended**
BCG																										
Hepatitis B																										
Polio																										
DTP-containing vaccine																										
Haemophilus influenzae type b																										
Pneumococcal	Conjugate																									
	Polysaccharide																									
Rotavirus																										
Meningococcal	MenA conjugate																									
	MenC conjugate																									
	MenACWYconjugate																									
	MenB																									
Measles, Mumps, Rubella																										
Measles, Mumps, Rubella, Varicella																										
Varicella																										
Hepatitis A																										
HPV	Nonavalent																									
	Quadrivalent																									
	Bivalent																									
Seasonal Influenza	Quadrivalent																									
	Trivalent																									
	Inactivated, standard-dose, egg-based																									
	Inactivated, standard-dose, cell culture-based																									
	Live attenuated, egg-based																									
	Inactivated, high-dose, egg-based																									
	Inactivated, standard-dose, egg-based with MF59 adjuvant																									
	Recombinant HA																									
Herpes zoster	Live, attenuated																									
	Recombinant, adjuvanted																									
Japanese Encephalitis																										
Yellow Fever																										
Tick-borne Encephalitis																										
Typhoid																										
Cholera																										
Rabies																										
Dengue (CYD-TDV)																										

Co-funded by
the Health Programme
of the European Union

Vaccine introduction / recommendations planned in the upcoming three years, by medical condition and other indications

Antigen		Immuno-compromised (excluding HIV infection)	HIV infection CD4 count		Kidney failure, end-stage renal disease, or on hemodialysis	Heart disease	Chronic lung disease	Alcoholism	CSF leak or cochlear implant	Asplenia or persistent complement component deficiencies	Chronic liver disease	Diabetes	Considerations	
			<200/mm³	≥200/mm³									Introduced	Recommended
BCG														
Hepatitis B														
Polio														
DTP-containing vaccine														
Haemophilus influenzae type b														
Pneumococcal	Conjugate													
	Polysaccharide													
Rotavirus														
Meningococcal	MenA conjugate													
	MenC conjugate													
	MenACWY conjugate													
	MenB													
Measles, Mumps, Rubella														
Measles, Mumps, Rubella, Varicella														
Varicella														
Hepatitis A														
HPV	Nonavalent													
	Quadrivalent													
	Bivalent													
Seasonal Influenza	Quadrivalent													
	Trivalent													
	Inactivated, standard-dose, egg-based													
	Inactivated, standard-dose, cell culture-based													
	Live attenuated, egg-based													
	Inactivated, high-dose, egg-based													
	Inactivated, standard-dose, egg-based with MF59 adjuvant													
	Recombinant HA													
Herpes zoster	Live, attenuated													
	Recombinant, adjuvanted													
Japanese Encephalitis														
Yellow Fever														
Tick-borne Encephalitis														
Typhoid														
Cholera														
Rabies														
Dengue (CYD-TDV)														

Co-funded by
the Health Programme
of the European Union

Antigen		Pregnancy	Health care personnel	Men who have sex with men	Other indications/ Special situations	
						Considerations
BCG						
Hepatitis B						
Polio						
DTP-containing vaccine						
Haemophilus influenzae type b						
Pneumococcal	Conjugate					
	Polysaccharide					
Rotavirus						
Meningococcal	MenA conjugate					
	MenC conjugate					
	MenACWY conjugate					
	MenB					
Measles, Mumps, Rubella						
Measles, Mumps, Rubella, Varicella						
Varicella						
Hepatitis A						
HPV	Nonavalent					
	Quadrivalent					
	Bivalent					
Seasonal Influenza	Quadrivalent					
	Trivalent					
	Inactivated, standard-dose, egg-based					
	Inactivated, standard-dose, cell culture-based					
	Live attenuated, egg-based					
	Inactivated, high-dose, egg-based					
	Inactivated, standard-dose, egg-based with MF59 adjuvant					
	Recombinant HA					
Herpes zoster	Live, attenuated					
	Recombinant, adjuvanted					
Japanese Encephalitis						
Yellow Fever						
Tick-borne Encephalitis						
Typhoid						
Cholera						
Rabies						
Dengue (CYD-TDV)						

Notes:
